# Double bookkeeping in schizophrenia spectrum disorder: an empirical-phenomenological study

**DOI:** 10.1007/s00406-023-01609-7

**Published:** 2023-04-21

**Authors:** Helene Stephensen, Annick Urfer-Parnas, Josef Parnas

**Affiliations:** 1 Center for Subjectivity Research, University of Copenhagen, Karen Blixens Plads 8, 2300 Copenhagen S, Denmark; 2 Mental Health Centre Glostrup, University Hospital of Copenhagen, 2605 Brøndby, Denmark; 3 Mental Health Centre Amager, University Hospital of Copenhagen, 1610 Copenhagen V, Denmark; 4 Faculty of Health and Medical Sciences, University of Copenhagen, 2200 Copenhagen N, Denmark

**Keywords:** Schizophrenia, Double bookkeeping, Psychosis, Insight, Reality, Phenomenology

## Abstract

Double bookkeeping is a term introduced by Eugen Bleuler to describe a fundamental feature of schizophrenia where psychotic reality can exist side by side with shared reality even when these realities seem mutually exclusive. Despite increasing theoretical interest in this phenomenon over the recent years, there are no empirical studies addressing this issue. We have, therefore, conducted a phenomenologically descriptive qualitative study of 25 patients with schizophrenia in which we addressed the following issues: (1) Experience of double reality; (2) Emergence and development of two realities; (3) Truth quality of psychotic or private reality; (4) Insight into illness; (5) Communication of psychotic experiences. The most important result was that most patients felt to be in contact with another dimension of reality. Hallucinatory and delusional experience pertained to this different reality, which patients most frequently kept separated from the shared reality. This other dimension was considered by the patients as being more profound and real. The pre-psychotic and psychotic experiences were difficult to verbalize and typically described as totally different than ordinary experience. Double reality was persistent across remissions. None of the patients considered their condition as an illness analogous to a somatic disorder. Most patients described a vague sense of duality preceding the crystallization of double bookkeeping. This emergence of doubleness was associated with a fundamental alienation from oneself, the world, and others stretching back to childhood or early adolescence. We discuss the results with a special emphasis on the concept of psychosis, clinical interview, treatment, and pathogenetic research.

## Introduction

Double bookkeeping is an important yet neglected feature of schizophrenia spectrum disorder. The term was first introduced by Eugen Bleuler in 1911 to capture the characteristic, although paradoxical phenomenon of schizophrenia where psychotic reality can exist side by side with shared reality even when these realities seem mutually exclusive [1]. One of our hospitalized patients, who believed that CIA had surrounded the hospital in order to kill him, nonetheless unconcerned left the hospital to buy ice-cream nearby. Professor Elyn Saks offers an articulate illustration of this double reality from a first-personal perspective:

[M]y life truly began to operate as though it were being lived on two trains, their tracks side by side. On one track, the train held the things of the ‘real world’—my academic schedule and responsibilities, my books, my connection to my family (. . .). On the other track: the increasingly confusing and even frightening inner workings of my mind. The struggle was to keep the trains parallel on their tracks, and not have them suddenly and violently collide with each other. [2]

In recent years, double bookkeeping has gained increasing attention in phenomenological and theoretical literature on the nature of delusions [3–8]. However, these studies mainly deal theoretically with the phenomenon and question whether it is adequate to view delusions as beliefs at all.

We recently published a paper on double bookkeeping based on long-lasting research, clinical experience with schizophrenia, and literature studies [9]. We claimed that the phenomenon comprehends a more global transformation of the experience of reality and phenomenal consciousness, which appears to be specific for the schizophrenia spectrum disorders. Rather than merely concerning delusions, double bookkeeping seems to be characteristic of most psychotic symptoms in schizophrenia and to manifest itself before the onset of overt psychosis in more subtle changes of the structure of subjectivity. The formation of a psychotic world seems to be associated with an alteration of the way of being in the world where the patient feels profoundly estranged from the world, others, and herself. The idea is that the original articulation of psychosis in schizophrenia consists in the emergence of an alarming openness to another presence within the patient’s most intimate subjective life. This is accompanied by a sense of breakthrough to some kind of “other” layer of reality varying from an inner life of quasi-solipsistic character (i.e., a sense to be the only existing consciousness) to contact with other-worldly dimensions. Patients often describe their psychotic reality as more true and profound than the socially accepted reality. To grasp this different layer of reality, we used the notion “ontological,” which refers to the nature of being as such, e.g., the structures of spatiality, temporality, or language. Importantly, this is a realm we do not ordinarily notice in our everyday lives, engaged in daily life activities, which is the so-called realm of the “ontic.”^1^ The idea is that psychotic experience by its ontological dimension touches upon different structures of meaning.

Since there are no systematic empirical studies on the issue of double bookkeeping, we decided to undertake a phenomenological-qualitative interview study of a group of patients with schizophrenia. In this report, we address the following issues.

1. Experience of double reality
2. Emergence and development of two realities
3. Truth quality of psychotic or private reality
4. Insight into illness
5. Communication of psychotic experiences

A closer comprehension of double bookkeeping may have a significant import for the understanding of the nature of psychosis, its management and treatment as well as conceptual issues in research on schizophrenia.

## Methods

### Sample

The patients were recruited from psychiatric services of the Capital Region of Denmark: Psychiatric Center Glostrup, Psychiatric Center Copenhagen, and Psychiatric Center Amager. All these services are affiliated with University of Copenhagen. The inclusion criteria comprised the diagnoses of schizophrenia spectrum (i.e., schizophrenia, other non-affective psychosis, and schizotypal disorder). The patients were required to be able to tolerate lengthy interviews, because the study targeted detailed qualitative aspects of experience. The exclusion criteria comprised organic brain disorder, IQ < 70, clinically dominating alcohol or substance abuse, acute/agitated condition, forensic status, or exposure to coercive interventions. The patients were contacted by their primary care staff and informed about the study.

In total 33 patients were contacted and 8 declined leaving the sample at 25 persons (8 males, 17 females, mean age 30.7 years; see Table 1). The reasons for decline comprised logistic problems, or lack of energy to use the time for the study (especially among outpatients who did not wish to make an extra trip to the outpatient clinic). One patient was excluded because of an overlooked forensic status. The inclusion diagnosis was the diagnosis made by the treating clinicians. However, all hospital charts were reviewed by the senior investigators (AUP, JP) in order to assure the fulfilment of the ICD-10 criteria. Upon this review 24 patients fulfilled the ICD-10 research criteria for schizophrenia and 1 patient for schizotypal disorder.

**Table 1:** Sociodemographic data

Gender (*n*)	Male	8
	Female	17
	Other	0
Age (years)	Mean (SD)	30.7 (11.3)
	Median (range)	26 (18–54)
Education	Primary school	8
	High school	7
	Completing high school	5
	University	1
	Completing university	4
Occupational status	Disability pension	7
	Unemployed	3
	Sick leave	7
	Actively studying or employed	8

Eight patients were recruited during hospital admission, whereas the remainder was recruited from outpatient clinics (*n* = 17). Six of these patients were recruited from an outpatient clinic for patients who had lived several years with schizophrenia, whereas 11 patients were recruited from an outpatient clinic for young patients with recent onset of psychosis.

### The interview

The interviewer (HS) is a philosophy PhD-fellow with four years of clinical experience as an employee at a psychiatric hospital where she had training in psychiatric interviews and the use of the Examination of Anomalous Self-Experience (EASE) interview [11]. AUP and JP are both senior consultants in psychiatry with clinical and research experience in the domain of schizophrenia. AUP participated in the majority of the interviews.

For this study, we prepared an interview guide according to phenomenological interview principles [12]. The interviews lasted between 1 and 4 hours and were sometimes split into two or more sessions. The interviews were semi-structured and conversational giving the patients ample possibility to describe their experiences. The structured element in the interview consisted in the obligation to cover the first four domains of the study outlined in the introduction. We used 15 items from the EASE interview focusing on the subject’s existential position, sense of basic self, and relation to the world and others (domain 1, 2, 4, and 5). We excluded domain 3 because of time concern. Domains 1, 2, and 4 are most specific to schizophrenia spectrum [13]. Domain 5 targeted existential issues and thus overlaps with the entire interview.

### Data analysis

All interviews were audiotaped and subsequently transcribed. The data analysis consisted in obtaining a consensus about the four target domains (top-down approach) following the principles of qualitative, thematic analysis [14]. The fifth domain related to the difficulties of verbalizing psychotic experience, which emerged during the analysis (bottom-up).

The patients participated on the condition of informed, written consent and the study was approved by the Data Protection Agency (P-2020-4), University of Copenhagen (514-0045/19-4000), and the ethics committee of University of Copenhagen.

## Results

We present the results divided into target sections of the interview.

### Experience of double reality

Most patients (*n* = 24) described a sense of existing in two realities. One reality being our shared, everyday world and the other reality being the world of private sometimes psychotic experience. In one case, it was not possible to ascertain the information needed for the assessment of the patient’s experience.

We found varieties of the experience of double realities that can overall be divided into two groups. The majority of patients (*n* = 18) described the psychotic reality as an insight or contact to a more true layer of reality (see

“Double reality: the second reality as an expression of another dimension”). The remaining patients (*n* = 6), although living in two realities did not ascribe any form of special insight or transcendent connection linked to the private reality (see “Double reality: the second reality as a private, quasi-solipsistic domain”). The two groups should not be seen as two sharply separated categories, but rather as representing different ends of a dimension.

#### Double reality: the second reality as an expression of another dimension

Patients described existing in two disjoint realities, namely the reality of the shared world and the reality of psychotic experience (i.e., hallucinations and delusions). The psychotic world was described as something behind or beyond the appearing, physical world often with terms like “mystical,” “supernatural,” “quasi-religious,” or the like. Hallucinations or delusions were considered as insight into or messages from a different dimension or parallel world.

*Case 8: “I’ve always lived in two parallel worlds.. Meaning that I live in the world everybody else does, where we know that the table is a table, and then in my own world, where I have visions and hear voices. But my sense of reality is intact. I know that you can’t see and hear what I can see. I can easily keep them apart.”*

*Case 27: “There is this common reality, that we share, and then I can tap into this other reality. It is some sort of understanding of how everything in the world is connected […] In the other world, I think there are some supernatural beings controlling the world and deciding how things are happening. Somewhat God-like. And I think everything is set up for me.”*

*Case 11: “I thought I was an alien from a faraway planet (…) I believe that there are several dimensions and that they are so close to each other that it is difficult to see the difference (…) I can feel a little difference, something strange, and then I think: ‘I wonder if I just entered another dimension?’ You can never be totally sure because the worlds look like each other. It’s not like the sky is suddenly pink.”*

#### Double reality: the second reality as a private, quasi-solipsistic domain

Some patients experienced double reality in the sense of feeling divided between their private world and the shared, external world (*n* = 6; the only patient with schizotypal disorder belonged to this group). They described their inner world with a quasi-solipsistic quality, i.e., a transient sense of being the center of the universe or that their experiential field was the only truly existing reality. They felt to exist or being locked inside their own heads. This inner world felt to exist side by side with the shared world in a disjoint manner rather than being in dynamic contact with it. The patients did not report explicit feelings of contact or insight into another dimension of reality.

*Case 20: “I live inside my own head (…) I know what is real and what is not real, but sometimes I get a little confused (…) It can be difficult controlling to [return to the real world], because sometimes I don’t know where I am (…) It’s not like I imagine that I’m in another dimension or that I exist in another physical world. It’s more something that goes on in my head.”*

*Case 16: “It is as if I live between two worlds. There is my own, little world and then there is the surrounding world. And I need to juggle between what I focus on and where I am present […] I spent most of the day being inside my own head rather than being in the real world. It takes a lot of time and energy to exist on two tracks at the same time.”*

*Case 21: “It feels like I have to fight my way out of a daydream and all the time remind myself to be present or to try to focus on something present, to become less out of tune [with everyone else] (…) Sometimes it keeps running in the background, even if I’m for example in the middle of eating dinner [with my family]. It’s like a movie that keeps running on the inside.”*

## Emergence and development of two realities

Most patients experienced a sense of double realities since childhood or early adolescence. It was often difficult for the patients to determine an exact time of emergence since it felt to be a habitual part of their experiential life. They associated the emergence of double realities with feelings of a fundamental estrangement from the shared world (see “The emergence of double realities: feelings of being different and derealization”), and their sense of self (see “The emergence of double realties: self-fragmentation”).^2^ The patients with recent onset of schizophrenia remembered more vividly the beginning of these experiences than patients in later stages of psychosis. Concerning the development and course of two realities over time, most patients described that the sense of two realities remained constant across the intensity of the illness (see “The course of double reality”).

### The emergence of double realities: feelings of being different and derealization

All patients described feeling profoundly alienated from the shared world in the sense of being fundamentally different from others (“*Anderssein*”), experiencing the shared world as unreal or somehow artificial (i.e., derealization), and a radical feeling of not truly belonging to the shared reality. The patients reported a profound sense of solitude and an unbridgeable distance from other people. Many patients associated this sense of being “outside” the shared world with feelings of being in a different world than others and a beginning sense of contact to this other world.

*Case 18: The patient, 18 years old, described the sense of two realities as emerging gradually over the course of many years, and it became explicit and persistent 1 year ago. She always had the sense to fundamentally exist “outside” the world and that other people were not authentic: “When you watch a movie, and the cameras act as someone’s eyes, that is how I feel. You see everything that goes on around you, but it doesn’t feel like you are present (…) It is like a film that just runs while you sit and watch, and you cannot really be part of it, but you can also not,* not *be part of it, because obviously you are there (…) your body and your surroundings are unreal, but your head is the only thing that exists and is really real, and then there is somewhere else, a place.” One day, out of the blue, the following thought emerged in the form of a voice: “They are in one world, and I am in another.” This voice is not experienced as the patient’s own voice, but feels like “contact” to another dimension.*

*Case 13: “It’s just a feeling that it is difficult to fit in with other people.. I don’t really know how to explain it but it’s just like there is something that kind of stands out.”*

*Case 28: “All that is visible of the iceberg is everything that you can observe.. as for example that one becomes psychotic and think that there is a lizard in the room (…) or get paranoid. But actually, I feel more sick in what happens in the iceberg below the surface of the water. This means a completely, concrete different* way *of perceiving the world than all other human beings (...) It is much more frightening to fundamentally feel that one is from another planet [than being in a psychotic state] (…) I feel profoundly emotionally distanced from other people because I feel that I have access to a different level of consciousness than others.”*

### The emergence of double realties: self-fragmentation

All participants reported self-alienating experiences pivoting around a fragile sense of their most intimate sense of existing as a subject, e.g., feelings of not truly existing, not being fully present, or an experiential distance to their own thoughts, feelings, or actions. In relation to the emergence of double realities, patients typically mentioned self-alterization (i.e., a pronounced, anonymous otherness in the middle of subjective life) and simultaneous introspection (i.e., involuntary self-monitoring disturbing the patient to fully engage in various activities such as social interaction or watching television). These experiences were associated with a sense of division of the patient’s own subjectivity between different realities or parts.

*Case 25: The patient feels divided between himself as an “individual” and himself as “a person in society.” He often questions “who is the true me?” He observes himself instead of being engaged in situations: “I become almost out of myself. I can observe myself existing (…) when I heard the squealing train tracks, I also heard the sound itself (…) and I became conscious that it felt like there was more in it than there maybe was (…) I felt there was a deep, inner voice that could observe (…) There is something rational, observing from the inside, simultaneously as there is the very thing that I experience or do.”*

*Case 26: The patient relates double bookkeeping to a sense of being “two persons.” “It feels like there is something inside your own self that you cannot relate to in your head (…) something that you cannot relate to, which is yourself. (…) Sometimes I am so much inside my head that I am without a body.”*

### The course of double reality

Most patients described a persisting sense of double reality with fluctuating salience of one of the two. They mentioned the periods where they felt mostly at ease as when there was a balance between the two. This implied that they could keep these two realities separated. In these periods they did usually not enact their psychotic experiences in the shared reality. However, occasionally and typically in acute psychotic exacerbations, the two realities collided and became confused with each other. In the phases leading to hospitalization, the psychotic or inner world typically became increasingly invasive and out of control. Many patients, while in their psychotic condition, were acutely aware of what was going on around them but had a difficulty in communicating this awareness. Importantly, during the remission, the significance of psychotic experiences remained intact.

*Case 27: A 29-year-old patient reported a “persistent feeling of another world” during the last 7 years. Previously, she experienced vague signs of this other world (e.g., she felt that other people were manipulating or controlling her and that things were staged). This other world was always there, also when she felt to “not exist in it.”*

*Case 15: The patient described that even when she felt that her psychotic experience was not true, the sense or significance of these experiences was nevertheless preserved: “It was a strong feeling. I think it can maybe be defined as a delusion, maybe you can call it that (…) Now, I can see that it makes no sense that my frontal lopes are made of starlight, but I still have a feeling deep inside, believing that this is the case.”*

## Truth quality of psychotic or private reality

The truth quality of psychotic (see “Truth quality of psychosis”) or private (see “Truth quality of private world”) reality was typically described as a different kind of truth than that pertaining to the shared world.

### Truth quality of psychosis

Most participants were able to distinguish their psychotic experience from daily life experience. Patients were aware of their hallucinations or delusions as private rather than intersubjectively valid. This awareness would not make patients question the truth of psychotic experiences in the sense of their importance, relevance, or meaningfulness. On the contrary, patients often described psychotic experience in terms of being more “real” than the “real reality” and as something involving a deeper level of truth, transcending common sense knowledge. It was not possible to doubt the certainty of these experiences. Typically, patients reported that the meaning involved in psychosis came from the outside with a revelatory character, arising suddenly in the middle of the intimate or affective sphere of their subjectivity. The meaning did not always have a specific content and was often enigmatic and puzzling for the patients themselves. Although the meaning was vague, the patients knew undoubtedly that it uniquely had something to do with them. Psychotic experience was described with a quality of being alien or unfamiliar compared to ordinary perception, thinking, or imagination, resembling perceptual experience without in fact being like it (e.g., “seeing without seeing”).

*Case 26: “[The parallel universe] has a very different quality because it is not something that melts into my daily life. (…) Psychotic experiences are extremely alien. It’s like if you are walking to your kitchen, open the door, and then it’s a different kitchen (…) It is physically impossible things happening (…) When I’m in a psychotic state I can in fact differentiate it from what it should be from a logical perspective. But when you are in the situation, it is extremely difficult to think logically because you see it, hear it, or feel it, and it is very difficult to contradict something that you can see.”*

*Case 24: Pt: “I used to think that I’m [the center of the universe]. It doesn’t sound good to say, but I thought that I was Jesus or that I was chosen to do something great.”*

*I: “Where do you know it from?”*

*Pt: “I don’t know. It is a truth like ‘gravity exists.’ I just know.”*

*Case 25: The patient reported a hallucinatory experience of seeing a woman. “It wasn’t like I physically saw something change, but it was more like a mixture, dream-like, but more visual somehow. I looked up and saw a woman figure standing at the top of a staircase (…) If I close my eyes and move my arm, I can sense how my arm is moving without seeing it. It feels like that.. It felt like it exceeded consciousness (…) It is like seeing without really seeing (…) It is there, but in the back of my head, inside the mind, not in my [physical] eyes.”*

### Truth quality of private world

Most patients described their inner world with a different truth quality than other aspects of their inner life and ordinary perceptual experience. This inner world was populated by daydreams and fantasies that, however, differed from normal imagination by acquiring a certain autonomy, and spatial characteristics. This had some type of affective and immediate truth value, sometimes more true than the shared reality. The private fantasy world felt closed off from the shared world in a radical manner as something uniquely involving the patient and without any dynamic interplay with the shared world. In contrast, other parts of the patients’ inner life were often described as too open, accessible, or transparent for others (i.e., transitivism). They felt to be both the sole creator of this universe and at the same time, a passive spectator. Furthermore, it was difficult or impossible to keep up with the inner and shared reality at the same time.

*Case 21: “When I say I don’t doubt what is real [and what is daydream], then it depends on what you mean by real. Because it has some sort of quality for me, when I daydream. But it doesn’t have a quality like the table. (…) I think [the daydream world] has an emotional reality - not an objective [reality]. It can* feel* true. (…) I think this is why it can be difficult to change between the two worlds, because if you are in one emotional reality, then you somehow have to twist and turn to join the rhythm everyone else is in.”*

*Case 13: The patient feels divided between a private, phantasy world and a public world. “What I make up in my own head has nothing to do with the public, real world […] it is what I think and feel that are easily accessible to others. My phantasy world is closed.”*

## Insight into illness

None of the patients accounted for their symptoms of schizophrenia as being comparable to an illness in the ordinary sense of the term. Eighteen patients considered their psychotic symptoms as signs from another dimension, parallel, or supernatural world, or insight into a more true level of reality. The remainder, although not considering psychotic symptoms as signals from another dimension, nonetheless considered their schizophrenia as an integral part of their person. All patients except one found their “illness” to contain positive aspects, whereof most patients mentioned creativity. Several patients feared that antipsychotic medication would rob them of their creativity and flatten out their rich inner life.

*Case 13: “I think there is a part of me that always will be schizophrenic, whereas somatic diseases most of the time will pass and be over with.”*

*Case 10: “Well, I don’t really know. ‘Schizophrenia?’ I’ve read some explanations and models of explanation of it. Both the official psychiatric diagnoses and explanations and it doesn’t really explain anything. So of course, I have turned to the alternative (…) There are the psychotic symptoms, and what is that? To see things that are seemingly not there, which other people do not see or experience. Well, I have done that for 17 years now (…) The mystical and the supernatural. It just exists. (…) I actually think that both the voices and the visions originate from the astral dimension. It just makes sense to think about it in that way because I can’t explain it in any other possible sense. (…) Anxiety, depressed thoughts, and pain, and those kinds of things are something one could consider illness.”*

*Case 3: The patient has multiple psychotic symptoms. The constant theme in his thinking is the idea that he is Jesus: “Now that I feel better, I know that [the idea that I’m Jesus] is a part of my illness*—*a delusion. But it created a whole atmosphere so I cannot help that other people still think that I’m Jesus.” Asked what he thinks about his schizophrenia diagnosis, he replied: “it seems quite true. All that with the split personality” I: “How so?” Pt: “When I’m happy, then I’m happy Jesus, when I’m sad, then I’m failed Jesus. And sometimes I’m just myself, when I’m on medication.”*

*Case 24: “When I feel bad, I think it is an illness (…) and then it’s nice to be able to say it is an illness because then it’s something beyond myself, but mostly it is difficult to call it ‘illness’ because it is me, and it’s not like putting plaster on your leg (…) it’s the very way my mind functions (…) if you call it illness you will think of it as an enemy or something that you need to get rid of.”*

## Communication of psychotic experiences

Most participants explicitly described difficulties in verbalizing their psychotic experience. Typically, they only disclosed their experiences to others after many years.

*Case 27: A 29-year-old patient experienced psychotic symptoms for nine years but only disclosed these during her second contact with psychiatry one year ago. In her first encounter with psychiatry eight years ago, she did not feel listened to. “I [was angry about] only seeing [the psychiatrist] one time and it was a questionnaire (…) There was no conversation about how I was doing, my life circumstances, etc. (…) I really needed to talk to someone and she [the psychiatrist] didn’t want to. She just wanted to diagnose me and get it done.”*

*Case 25: “It [hallucinatory experience] felt as if it exceeded consciousness, like it ‘bubbled over.’ You can no longer describe it, because it is so.. it was so.. it was so.. so wild and it was so beyond, it was so beyond (…) It’s extremely difficult to describe (…) like a pure sensing without logical thought.”*

*Case 26: “I don’t really know how to formulate it [psychotic experiences], the only word I can think of is “supernatural,” but it’s not really that. It’s very alien.”*

## Discussion

In the following, we will first address the methods and limitations of the study and then discuss the significance of our results separated into the overlapping sections: (1) the double in double bookkeeping: beyond the question of reality; (2) insight into illness; (3) the emergence of double realities: self-fragmentation and *Anderssein*; (4) communicating psychotic experiences. This overlap is unavoidable, because double bookkeeping is not an isolated symptom but expressive of a specific change in the structure of subjectivity.

### Methods and limitations

A key methodological challenge is that double bookkeeping is a phenomenon that pervades multiple aspects of experience, cognition, and behavior. Thus, the study involved in-depth, narrative interviews, and a subsequent time-consuming analysis involving the three authors. Given these difficulties, the sample size appears reasonable for a qualitative study of this type. We cannot be certain that the selected patients are representative of schizophrenia in an epidemiological sense, but we believe that our mixture of patients with recent onset of psychosis and advanced patients is comparable to patients with schizophrenia in general. It is important to note that none of the patients was in acute psychosis or a severe exacerbation of their illness.

### The double in double bookkeeping: beyond the question of reality

From a phenomenological perspective, double bookkeeping is not simply a reflection of holding conflicting attitudes, beliefs, or perceptions. Rather, the delusional and shared reality can exist side by side without conflicting because these realities are incommensurable [5, 9, 15]. Jaspers termed the apparent incongruence between action and the content of a delusion as “inconsequential attitude” [16, 17].

The participants in our study did typically not experience any contradiction in the sense of incompatibility. Rather, they experienced the two realities as separate domains that are rarely confused. This means that the two realities are not simply different but that they cannot be judged by the same standard. As most participants reported, psychotic experience has a completely different quality than ordinary experience (e.g., the mode of givenness is characterized by hyperproximity, because it happens in the midst of the subject). This is in line with the findings in recent phenomenological-empirical studies [18–21].

Now, the question is what this other realm of reality more precisely means? For a minority of patients, the other reality consists of an enclosure in a purely immanent, subjective life that is often solipsistically tainted and cut off from a dynamic exchange with the shared, social environment. The majority of patients reported an access to a dimension of reality hidden for others. Psychotic experience is distinguished from ordinary experience as it seems to be also concerned with a realm beyond the sensory. A patient described it as a truth “behind all appearance.” Others compared it to mystical-like, other-worldly, or divine experience (see also [22]). Importantly, these experiences are imbued with a sense of absolute certainty (as apodictic truths), which precedes any specific *content* of delusional or hallucinatory experience [23]. In other words, the affective moment of experience precedes its cognitive elaboration. A patient described a paranoid fear as a feeling that anteceded a specific content of that fear: “It was like the fear was already there from the inside and then it found its target.” This sense of certainty is different from everyday perception. Phenomenologically, the latter is imbued with doubt, or more precisely the possibility to be corrected by interaction with one’s surroundings [24]. The affective certainty of psychotic experiences is associated with another important feature, namely that these are profoundly singular and subjective. Patients describe the experiences as something uniquely concerning them. In sum, psychotic experience transcends the sensory and shared reality and does not seem to be integrated or “woven into the fabric of the intersubjective world” [15]. It is crucial to emphasize that this does not mean that psychotic experience is simply “outside” the shared reality in the sense of being completely unrelated to it. Rather, psychosis concerns a different ontological *layer* of reality, namely the very meaning or nature *of* reality. As one of our patients explained, she often struggled to grasp *what* people were saying because she started to think about the very *meaning* and *truth* of language. We can paraphrase Müller-Suur’s observation that the alteration of experience in schizophrenia concerns the “horizon of meaning” (“*Sinnhorizont*”) [25]. The same empirical object can be regarded upon different horizons of meaning, e.g., as something pragmatically useful, as something created, a sacred item, or an exemplar of materiality of the world. In a similar vein, Blankenburg pointed to an alteration of contextual framework of experience, rather than to a change in the content of experience [26]. When patients question the context or validity of reality, it is different from questioning if something is real or not in the standard sense of the term. The latter often leading to misunderstandings between patients and clinicians. The phenomenological point is that when we perceive something, we also implicitly and tacitly perceive a whole network of significance and a familiarity within a given intersubjective framework. Briefly put, the other layer of reality involved in psychosis may pertain to the axioms or structure of reality (ontological level). The two realities involved in double bookkeeping can thus be incommensurable, although they concern one and the same reality. In the face of this, many patients described a sentiment of being split or divided. It could, therefore, perhaps be more precise to speak of a rupture within reality rather than double reality. Rather than being two separate perceptions or beliefs, double bookkeeping is expressive of a specific “unified divided consciousness” as we phrased it elsewhere [9]. It makes no sense for the patients to speak of their psychotic experience as true or false by empirical or mundane standards and it is not possible to prove (logically or empirically) that a given delusion or hallucination is incorrect. The idea is that psychosis does not primarily concern the sphere of reason (judgment or perception), but rather an alteration of the structure of subjectivity in its basic, pre-cognitive relation to the shared world.

### Insight into illness

Our results, especially the tendency of persistence of psychotic reality between so-called “relapses” is consistent with the findings of Jones & Shattell [27]. Thus, the notion of “a psychotic episode” is often not valid for the course of schizophrenia. Briefly put, double bookkeeping begins to emerge early in life and may become a persistent condition. As we have already mentioned, the patients do typically not consider their psychotic experiences as an expression of illness, but rather as constant companions that they need to keep apart from their interactions in the social world. The participants were likely to consider depression, anxiety, lack of energy, and initiative as signs of illness. This finding is consistent with studies showing that first-contact with treatment facilities is motivated by these so-called non-specific symptoms, rather than complaints about psychosis [28]. As already noted, the patients do not consider their experiences as pathological but as phenomena testifying to their access to another domain or level of reality. In mainstream psychiatry, the insight into illness is defined as an awareness of the illness, its symptoms and signs, risk factors, consequences, and the need of treatment. This medical definition implies an experiential distance between the self and symptoms. In the case of schizophrenia, the patients have no possibility for such an experiential distance, because psychotic phenomena originate in the intimacy of their own selfhood and, therefore, carry with them an apodictic certainty. This is the case notwithstanding the fact that psychosis often inflicts a severe suffering. As Mørck expressed it in her first-person account of living with schizophrenia: “I am 46 years old today, and I do not believe in the word ‘recovery’ […]. I coexist with schizophrenia, and it is as big a part of my identity, as part of me dealing with the outer world” [29]. In sum, when participants do not regard their psychotic symptoms as illness it does not seem to reflect poor insight, but rather to reflect double bookkeeping. Many patients have a double-awareness as it is well illustrated by the philosopher Wouter Kusters accounting for his first-personal experience of psychosis:

For me, that was beyond strange. I knew exactly what a psychosis was–I was right in the middle of one–and yet I couldn’t pull myself out. The psychosis presented itself to me as an inescapable truth and reality. [30]

### The emergence of double realities: self-fragmentation and anderssein

Double bookkeeping is not a contingent feature of schizophrenia, but rather an expression of its core *Gestalt*. Our patients described some sort of transformation of their existential position (basic relation to self, world, and others), including a feeling of being fundamentally different from others (*Anderssein).* This alteration of the self-world-relation can be either emphasized on the side of the subject or in its relation to the surrounding world and others [31, 32]. On the purely subjective level, there is a self-fragmentation (self-alterization), which consists of the parts of the subject acquiring an alien otherness: “It feels like there is something inside your own self that you cannot relate to in your head.” These alien fragments constitute the kernels upon which the other reality progressively articulates itself and eventually becomes the stage for the psychotic phenomena. Phenomenologically, we can describe this as a fragile sense of basic self or first-person perspective. The first-person perspective implies that all my experiences are given to me as my own, as *my* experiences [33]. I do not need to ask myself if it is me who is now looking at my computer screen. In other words, all experience involves a tacit self-affection (“auto-affection”) [9]. My experiences are self-saturated, shot through by a dimension of a tacit affective self-presence. However, this basic self is not an undifferentiated homogeneity but is a dynamic structure of diverging and coalescing affective moments. Subjectivity is open to the world and is always given to itself in this relatedness, affected by something other than itself and thus involving a structural, potential alterity. It seems that in schizophrenia, the moments of alterity become unintegrated or congealed, leading to the formation of intrusive, alien otherness, i.e., self-alterization or self-fragmentation [34]. Thus, this change of subjectivity is highly correlated with an altered relation to the world and others.^3^ The basic vulnerability of schizophrenia implies a breach in the dynamic with the shared reality. Minkowski described this alteration as a “loss of vital contact with reality” [35] and Blankenburg designated it as a “crisis of common sense” [36]. It is an alteration of pre-reflective and pre-conceptual grasp of intersubjectively and contextually valid meanings resulting in an enigmatic and often threatening coloring of the world. The majority of our patients described this progressive self-alienation and alienation from others and the world as beginning already in childhood or early adolescence and apparently functioning as a precursor of the crystallization of double bookkeeping. Briefly put, we see elements of double bookkeeping before the development of frank psychosis. Psychotic experience takes place within the intimacy of the patient’s subjectivity and simultaneously feels exterior, which gives rise to the sense of a rupture within reality. It is important to emphasize that the idea that the ‘other reality’ originates in the middle of subjectivity does not exclude a developmental or intersubjective aspect of psychosis [37, 38]. In many cases, patients described their psychotic experiences as something giving the patient a meaningful subjective position in the universe (see also [39]). Furthermore, patients described their private or psychotic world as a place where they felt at a safe distance from the unpredictability and ever-changing character of shared reality. It is important to note that the emergence of double realities should not simply be understood as a coping strategy. Rather, it is a “phenomenological compensation,” i.e., not as a willed or intentional act on the part of the patient, but rather as an automatic re-organization of consciousness as a way to remain in contact with reality or preserving a sense of existing as a subject [40].

### Communicating psychotic experiences

It is crucial to discuss the difficulty for the patients to verbalize these subtle phenomena and for the clinician to help patients to report them. First, it requires of the clinician to be attuned to the patient in a specific way, i.e., to let the patients unfold their self-descriptions without judgmental interruptions and premature categorizing. The latter requires a broad knowledge of psychopathological phenomena that are not yet converted into categorical symptoms [16]. Surprisingly, studies show that even trained psychiatrists are not always capable to facilitate self-descriptions of the patients. On the contrary, even when patients actively tried to talk about their psychotic symptoms, the psychiatrists avoided further exploration [41, 42]. Secondly, it is difficult for most persons to respond to questions of how they experience reality, how their thoughts feel like, and so forth. Most participants mentioned explicitly the difficulties of finding the right words for the psychotic experience. A patient articulates that the other reality is “some sort of understanding of how everything in the world is connected.” Thus, rather than involving a specific content, it involves a change in the very mode of experiencing and meaning. In other words, communicating this experience is difficult, because it concerns a realm outside of ordinary experience, language, and rules of logic. The patients often use metaphors, which may be sometimes shifted into a private use of words. We have not found any psychiatric studies that are explicitly concerned with the relation between pre-verbal experience and its expression in language. However, already in 1914, a French psychopathologist emphasized that in psychosis a central problem consists in the patients having experiences, which cannot be framed in an intersubjective discourse [43].

## Conclusion and implications

The literature on double bookkeeping portrays it as paradoxical since patients appear to hold self-contradictory beliefs as in the prototypical example of the patient who gladly consumes poisoned food. This self-contradiction made Bleuler question whether the patients regarded their delusions as real or not, which to this day is a frequent concern of clinicians. However, this question of reality when it comes to psychosis seems to be misguided. If you ask the patients whether they think their psychotic experience is real or not it is nonsensical from the patients’ perspective. It would be like asking someone with a toothache whether they believe the pain is real or not. Therefore, we argue that psychosis is not a question of real or not, but rather a question of reorganization of subjectivity and the meaning of reality. Consequently, we believe that the primary disturbance is located on the level of experience and affectivity rather than on the level of cognition.

We believe that double bookkeeping is an integral dimension of the schizophrenia *Gestalt,* involving alterations of selfhood and intersubjectivity. It is thus specific for the schizophrenia spectrum disorders. The awareness of this phenomenon is crucial in the interaction with patients with schizophrenia. The symptomatic picture of schizophrenia cannot be regarded on analogy with somatic illness where symptoms and signs are often well-delimited objective entities with referential function pointing to underlying pathology of the substrate. In schizophrenia, the psychotic phenomena have no referential function but are a configuration of altered structure of the subject’s being-in-the-world. We would like to emphasize that our qualification of the inadequacy of the medical model is not motivated by any romantic version of schizophrenia but by a concern for adequate treatment and research. The phenomenon of double bookkeeping has consequences for the nature of clinical examination, which today has become simplified to checklists or structured interviews that are not designed to elicit and comprehend this experiential alteration.

The notion of psychosis, which is basically undefined in contemporary psychiatry, heavily relies on the detection of delusions and hallucinations. These are considered as false beliefs and false perceptions where the patient is unaware of their falsity. However, as our and other studies indicate, this is very frequently not the case. In other words, assessment of psychosis requires a more refined psychopathological exploration and description than the commonsense notion of “falsity.” Moreover, the phenomenon of double bookkeeping questions the view of schizophrenia as a series of relapses and remissions of psychosis. Perhaps, it would be more appropriate to speak of exacerbations, because the change of subjectivity appears to have a tendency to persist. With respect to treatment and psychotherapy, it is most important to help the patient negotiate a balance between the two realities and prevent the exacerbations where the psychotic world overwhelms the patient and translates into severe suffering or maladaptive behaviors [44]. Finally, with respect to pathogenetic research, it is perhaps more important to focus on the phenomena of subjectivity rather than studying neuroscientific correlates of multifarious psychotic symptoms. More specifically, we believe that pathogenetic research can take advantage of a more refined psychopathology.

## Footnotes

1 The terms ontic and ontological are technical philosophical notions, which we will not discuss in detail in this paper [10].

2 This division should be seen as a pedagogical move because in most cases these two experiential domains seemed to be interdependent.

3 In terms of the EASE scale, the patients typically reported diminished sense of basic self, distorted first-person perspective, and loss of thought ipseity, as well as a sense of I-split. Furthermore, patients reported derealization, hyperreflectivity, loss of common sense/perplexity/lack of natural evidence, transitivism, and solipsistic-like experiences.
